# Bell's Palsy: A Prospective Study

**DOI:** 10.1155/2020/2160256

**Published:** 2020-03-16

**Authors:** Ahmed Hassan Kamil Mustafa, Ahmed Mohammed Suleiman

**Affiliations:** ^1^King Saud Bin Abdulaziz University for Health Sciences, Department of Maxillofacial Surgery and Diagnostic Sciences, College of Dentistry, Riyadh, Saudi Arabia; ^2^University of London, FFDRCSI, London, UK; ^3^Department of Maxillofacial Sugery, College of Dentistry, University of Khartoum, Khartoum, Sudan

## Abstract

**Background:**

Bell's palsy is an acute idiopathic facial nerve paralysis of sudden onset. It is the most common cause of lower motor neuron facial nerve paralysis with an annual incidence of 15–30 per 100,000 population. The objective of this work is to study the grade of the attack and the associated symptoms of Bell's palsy in a group of Sudanese patients. The study type is an analytical prospective-based study. The study was carried out at Khartoum Teaching Dental Hospital, Khartoum General Teaching Hospital. In this prospective of the study, 48 patients with Bell's palsy were evaluated using the House–Brackman scale in relation to the above mentioned variables.

**Results:**

The study showed 18 patients (37.5%) were grade II, and 24 patients (50%) had postauricular pain before and during the attack. By the end of the study period, 40 patients recovered completely (83.3%), and 8 (16.7%) patients did not recover completely, 5 (10.4%) patients complained of hearing changes during the attack, and 13 (27.1) patients gave a history of exposure to cold before the attack.

**Conclusion:**

Based on our prospective study, we conclude that the percentage of complete recovery decreases with increased severity of the attack at onset. We failed to demonstrate any relation between postauricular pain and prognosis of Bell's palsy. The percentage of taste changes in our study is low in comparison with those obtained in the literature. In addition, all the patients showed complete regain of taste sensation. The percentage of patients with hearing changes in our study is high compared with some studies. In literature, we have no explanation for that, and it may be related to severity of the attack. In the present study, we found a strong association between exposure to cold and development of Bell's palsy. As the number of patients in our study is small and there is a limited period of follow-up, the study may not reflect the real situation; therefore, we need a large population-based study.

## 1. Introduction and Background

Bell's palsy is an acute idiopathic peripheral facial nerve paralysis of sudden onset and accounts for approximately for 75% of acute facial nerve paralysis [1].

Facial nerve paralysis has been known since ancient times by the Egyptians, Greeks, Romans, Incas, and other native cultures [2].

The first medical studies of the disease should be attributed to Avicenna [3]. He was the first to record the differences between central and peripheral facial paralysis.

Although the name of Sir Charles Bell, who published his findings in 1821, is usually associated with this condition, there are two papers, one published by Niclaus A. Friedrich in 1798 and the other by Richard Powell in 1813, whose observation of onset, physical findings, natural history, and recovery preceded those of Charles Bell [4].

Acute idiopathic peripheral facial palsy is a common disease with an annual incidence of 15–30 per 100,000 population [5]. Most patients recover completely, but about 15–30% are reported to be left with different degrees of sequelae [5, 6]. There are many possible causes of Bell's palsy, but still the aetiology remains obscure [5]. The term Bell's palsy should be restricted to idiopathic facial palsy.

Various reasons account for the scanty information on the incidence of Bell's palsy. Different specialties are involved in the management of patients with Bell's palsy; patients do not look for treatment as the condition is painless and frequently limited, or of short duration [7].

The epidemiology of acute idiopathic peripheral facial palsy has been discussed in several articles, sometimes with contradictory findings [8].

The only published study in the Sudan was a case report of Bell's palsy in seven Sudanese children, by Abbas and Prabhu in 1981 [9, 10], and our study (the epidemiology of Bell's palsy in Sudan) in 2018 [10].

This study is the prospective part of our study (the epidemiology of Bell's palsy in Sudan) [10], where the epidemiology, associated risk factors, and management were evaluated.

The purpose of this study is to evaluate the clinical aspects of Bell's palsy among Sudanese patients.

The House–Brackman grading system, which was used in this study, is the standard adopted by the FND Committee of the American Academy in 1985 and remains the most widely used facial nerve grading system [11].

### 1.1. Objectives

#### 1.1.1. General Objectives

To study the clinical presentation of Bell's Palsy in Sudanese patient's samples.

#### 1.1.2. Specific Objectives

The specific objectives are as follows:

- To determine the grade of the attack at onset
- To identify the various associated symptoms
- To identify the percentage of completely recovered and incompletely recovered patients

## 2. Patients and Methods

### 2.1. Study Design

This prospective study was carried out in Khartoum Teaching Dental Hospital and in both the Physiotherapy and Neurology Departments of Khartoum Teaching General Hospital.

The study involved patients with Bell's palsy attending Khartoum Teaching Dental Hospital and Khartoum Teaching General Hospital (physiotherapy and neurology department) in the period from 20/12/2009 to 20/03/2010. Patients will be evaluated using the House–Brackman scale during presentation and during recovery in addition to questionnaire and studied in relation to the following:

  The grade of the attack at onset
  The various symptoms associated
  Recovery

### 2.2. Inclusion Criteria

Patients affected by acute onset facial paralysis without detectable cause.

### 2.3. Exclusion Criteria

  Known traumatic, inflammatory, and neoplastic pathology of the facial nerve in its intra- or extracranial course
  Bilateral facial paralysis
  Concurrent disease of the central or peripheral nervous system

### 2.4. Sample Size

Forty-eight patients diagnosed as Bell's palsy in Khartoum Teaching Dental Hospital and in Khartoum Teaching General Hospital (physiotherapy and neurology departments) were selected and followed up during the period from 20/12/2009–20/3/2010.

### 2.5. Data Analysis

The data were analysed using Statistical Package of Social Sciences (SPSS) version 15.

### 2.6. Ethical Consideration

An ethical clearance has been obtained from the ethical committee of the Faculty of Dentistry (U of K) and the ethical committee (MOH), and an informed consent has been signed by participants after explaining the objectives of the study.

## 3. Results

### 3.1. The Grade of the Attack at Onset

Of the 48 patients with Bell's palsy, 18 (37.5%) patients were grade II at onset, 7 (14.6%) patients were grade III at onset, 15 (31.3%) patients were grade IV at onset, 7 (14.6%) patients were grade V at onset, and 1 (2.0%) patient was grade VI at onset.

### 3.2. Associated Symptoms

#### 3.2.1. Postauricular Pain

Of the 48 patients with Bell's palsy, 24 (50%) patients had postauricular pain before and during the attack, and 24 (50%) patients did not complain of postauricular pain before or during the attack.

#### 3.2.2. Hearing Changes

Of the 48 patients with Bell's palsy, 5 (10.4%) had hearing changes (phonophobia) during the attack, while 43 (89.6%) did not complain of hearing changes.

#### 3.2.3. Taste Changes

Of the 48 patients with Bell's palsy, 6 (12.5%) patients had taste changes (decreased taste sensation) during the attack, while 42 (87.5%) patients had no taste changes.

#### 3.2.4. Tearing Changes

Of the 48 patients with Bell's palsy, 10 (20.8%) patients had tearing changes during the attack, 38 (79.2%) patients had no tearing changes during the attack.

## 4. Exposure to Cold

Of the 48 patients with Bell's palsy, 13 (27.1%) gave a history of exposure to cold, while 35 (72.1%) patients did not give a history of exposure to cold.

## 5. Recovery

Of the 48 patients with Bell's palsy, 40 (83.3%) patients recovered normal function, while 8 (16.7%) patients did not return to normal function till the end of the follow-up period.

## 6. Discussion

### 6.1. The Grade of the Attack at Onset

It was not easy to apply the House–Brackman scale on our patients, particularly when distinguishing between grade IV and grade V onset. However, patients who presented with a clear grade II and III at onset accounted for 37.5% and 14.6% of the cases, respectively, while patients who presented with a grade IV onset accounted for 31.3% of the series, as shown in Table 1.

Pietersen [6], using his grading scale (Pietersen grading scale), found that 12% of his patients were grade II (slight paralysis) on presentation, and 13% were grade III (moderate paralysis on presentation) and only 4% were grade IV and V (moderately severe and severe).

In our study, the percentage of grade II (37.5%) and grade IV (31.3%) is high in our study, and the high percentage of grade IV may be related to late presentation of patients, which may be due to ignorance.

### 6.2. Associated Symptoms

#### 6.2.1. Postauricular Pain

As illustrated in Table 2, 50% of our patients complained of postauricular pain before and during the attack of Bell's palsy. In his study, Pietersen [6] found 52% of his patients suffering from postauricular pain and concluded that patients with postauricular pains have a significantly worse prognosis than those without pains [6]. Katusic et al. [7] studied the different prognostic factors and found pains other than that of the ear which had a significant relationship with incomplete recovery.

In the present study, we failed to demonstrate any relation between the postauricular pain and the prognosis of Bell's palsy in our patients, as 95.9% of them showed complete recovery, as shown in Table 3. The remaining 4.1% did not recover from the paralysis until the end of the three-month follow-up.

#### 6.2.2. Hearing Changes

As shown in Table 4, 10.4% of our patients had an associated hearing changes, which appeared to be high when compared to 5.4% reported by El Ebiary from Egypt (sample size: 580 patients) [12]. We have no explanation for that but may be related to the severity of the attack at onset.

#### 6.2.3. Taste Changes

Table 5 shows that, around 13% of our patients suffered from taste changes during the attack. El-Ebiary [12] found 14% of the patients in his study had a complaint of taste changes, while Pietersen [6] reported that 83% of the patients had partially reduced or abolished taste and that 80% regained normal taste function. The percentage of taste changes in our study is low in comparison with those obtained in the literature. In addition, all the patients showed complete regain of taste sensation, as shown in Table 6.

#### 6.2.4. Tearing Changes

Table 7 in this study showed that 20.8% of our patients had increased tearing during the attack (due to loss of function of musculus oribcularis oculi which prevents tears from being transported to the lacrimal sac). The same observation was reported by Pietersen [6] mounting up to 67% of his patients. The author also observed that 4% of the patients complained of dry eyes. The latter observation was not noticed among our patients probably because of the small number of cases studied.

### 6.3. Exposure to Cold

Exposure to cold has been condemned for Bell's palsy for a long time. El-Ebiary [12] found 5.4% of the patients gave a history of exposure to cold and concluded that this provides strong evidence against refrigeration theory of Bell's palsy. Danielides et al. [13] from Greece failed to demonstrate any significant relation between cold weather as a predisposing factor for the development of Bell's palsy. In the present study, we found a strong association between exposure to cold and development of Bell's palsy, as 27% of our patients gave a history of exposure to cold before the attack, which is shown in Table 8.

### 6.4. Recovery

Following treatment, 83.3% of the patients in this study regained normal function during the follow-up period, as shown in Table 9. On cross tabulation of grade at onset and recovery (Table 10), patients with grades II and III showed 100% recovery during the follow-up period (three months). Patients with grade IV at onset showed 86.7% recovery, and patients with grade V showed 8.6%, while no patient with grade VI paralysis showed complete recovery during the study period. It can be noticed that the percentage of complete recovery decreases with increased severity of the attack at onset.

Engstrom et al. [14] concluded that patients with a high degree of nerve degeneration at both the initial examination and the first follow-up have a poorer prognosis. Gordana and Stojanka [15] showed that an incomplete paralysis at the onset can have complete recovery at the end, while when there is complete paralysis at the onset, the end result after the follow-up period was disappointing, showing a degree of permanent paralysis.

## 7. Conclusions

Based on our prospective study, we conclude that the percentage of complete recovery decreases with increased severity of the attack at onset. We failed to demonstrate any relation between postauricular pain and prognosis of Bell's palsy. The percentage of taste changes in our study is low in comparison with those obtained in the literature. In addition, all the patients showed complete regain of taste sensation. The percentage of patients with hearing changes in our study is high compared with some studies. In literature, we have no explanation for that, and it may be related to severity of the attack. In the present study, we found a strong association between exposure to cold and development of Bell's palsy. As the number of patients in our study is small and there is a limited period of follow-up, the study may not reflect the real situation; therefore, we need a large population-based study.

## Figures and Tables

**Table 1:** The grading of the attack at onset among patients with Bell's palsy in KTDH and KTH.

Grade	Frequency	Percentage
Grade II	18	37.5
Grade III	7	14.6
Grade IV	15	31.3
Grade V	7	14.6
Grade VI	1	2.0
Total	48	100

**Table 2:** Percentage of postauricular pain among patients with Bell's palsy in KTDH and KTH.

Postauricular pain	Frequency	Percentage
Yes	24	50
No	24	50
Total	48	100

**Table 3:** Recovery among patients with Bell's palsy with associated postauricular pain in KTDH and KTH.

Recovery in patients with postauricular pain	*N*	Percentage
Complete recovery	23	95.9
Incomplete recovery	1	4.1
Total	24	100

**Table 4:** Hearing changes among patients with Bell's palsy in KTDH and KTH.

Hearing changes	Frequency	Percentage
Yes	5	10.4
No	43	89.6
Total	48	100

**Table 5:** Taste changes among patients with Bell's palsy in KTDH and KTH.

Taste changes	Frequency	Percentage
Yes	6	12.5
No	42	87.5
Total	48	100

**Table 6:** Regain of taste among patients with Bell's palsy in KTDH and KTH.

Patients with taste changes	*N*	Percentage
Complete regain of taste	6	100
Incomplete regain taste	0	0
Total	6	100

**Table 7:** Tearing changes during patients with Bell's palsy in KTDH and KTH.

Tearing changes	Frequency	Percentage
Yes	10	20.8
No	38	79.2
Total	48	100

**Table 8:** Exposure to cold among patients with Bell's palsy in KTDH and KTH.

Exposure to cold	Frequency	Percentage
Yes	13	27.1
NO	35	72.9
Total	48	100

According to Chi-squared test, the *p* value was 0.01.

**Table 9:** Recovery among patients with Bell's palsy in KTDH and KTH.

Recovery	Frequency	Percentage
Complete recovery	40	83.3
Incomplete recovery	8	16.7
Total	48	100

**Table 10:** Correlation between grade at onset and recovery of patients with Bell's palsy in KTDH and KTH.

Grades		Complete recovery	Incomplete recovery	Total
Grade II	Count % within grades % within follow-up	18 100% 45%	0 0% 0%	18 100% 37.6%

Grade III	Count % within grades % within follow-up	7 100% 17.5%	0 0% 0%	7 100% 14.6%

Grade IV	Count % within grades % within follow-up	13 86.7% 32.5%	2 13.3% 25%	15 100% 31.3%

Grade V	Count % within grades % within follow-up	2 28.6% 5%	5 71.4% 62.5%	7 100% 14.6%

Grade VI	Count % within grades % within follow-up	0 0% 0%	1 100% 12.5%	1 100% 2.0%

Total	Count % within grades % within follow-up	40 83.3% 100%	8 16.7% 100%	48 100% 100%

Chi-squared test, grade *∗* recovery: Pearson Chi-square: 25.234, asymp. sig (p value).

## Data Availability

The data used to support the findings of this study are included within the article.
